# Nurses’ Perspectives on an Electronic Medication Administration Record in Home Health Care: Qualitative Interview Study

**DOI:** 10.2196/35363

**Published:** 2022-04-22

**Authors:** Sara Karnehed, Lena-Karin Erlandsson, Margaretha Norell Pejner

**Affiliations:** 1 School of Health and Welfare Halmstad University Halmstad Sweden

**Keywords:** e-health, eHealth, eMAR, electronic medication administration record, homecare nurses, home health care, nursing profession, delegation, task-shifting, medication administration

## Abstract

**Background:**

eHealth is considered by policy makers as a prerequisite for meeting the demands of health care from the growing proportion of older people worldwide. The expectation about what the efficiency of eHealth can bring is particularly high in the municipal home health care sector, which is facing pressure regarding resources because of, for example, earlier discharges from hospitals and a growing number of patients receiving medications and treatments at home. Common eHealth services in home health care are electronic medication administration records (eMARs) that aim to communicate delegated tasks between professionals. However, there is an extensive gap in the research on how technology affects and is experienced by home health care professionals.

**Objective:**

The objective of this paper is to shed light on how home care nurses experience eMARs in a Swedish municipality.

**Methods:**

This qualitative interview study was conducted among home health care nurses using eMARs to facilitate communication and signing of delegated nursing tasks. The analysis of the interviews was performed using constructivist grounded theory, according to Charmaz.

**Results:**

Of the 19 day-employed nurses in the municipality where an eMAR was used, 16 (84%) nurses participated in the study. The following two categories were identified from the focus group interviews: nurses become monitors and slip away from the point of care. The nurses experienced that they became monitors of health care through the increased transparency provided by the eMAR and the measurands they also applied, focusing on the quantitative aspects of the delegated nursing tasks rather than the qualitative aspects. The nurses experienced that their monitoring changed the power relations between the professions, reinforcing the nurses’ superior position. The experience of the eMAR was regarded as transitioning the nurses’ professional role—away from the point of care and toward more administration—and further strengthened the way of managing work through delegation to health care assistants.

**Conclusions:**

Previous analyses of eHealth services in health care showed that implementation is a complex process that changes health care organizations and the work of health care professionals in both intended and unintended ways. This study adds to the literature by examining how users of a specific eHealth service experience its impacts on their daily work. The results indicate that the inscribed functions in an eHealth service may affect the values and priorities where the service is in use. This presents an opportunity for future research and for health care organizations to assess the impacts of specific eHealth services on health care professionals’ work and to further examine the effects of inscribed functions in relation to how they may affect actions and priorities at individual and organizational levels.

## Introduction

### Background

The extensive use of eHealth has brought many new services to health care organizations, such as electronic health records (EHRs), mobile health apps, and electronic medical records. As defined by Eysenbach [1], eHealth refers to the use of internet technologies to organize and deliver health care, aiming to improve health care by using information and communication technology. Owing to their many affordances, caregivers introduce eHealth services with the expectation of increasing patient safety, making care more effective, reducing costs, and providing better conditions for people to receive medical care at home [2-4]. Policy makers consider eHealth as a prerequisite for meeting the demands of health care from the growing proportion of older people worldwide [5], and national policies aimed at facilitating the progress of eHealth infrastructures have been adopted in many countries. In Sweden, the government has undertaken a political vision for the country to become a world leader in using the opportunities offered by eHealth; therefore, an agency has been established to support this process [6]. The expectations of what eHealth can bring are particularly high in the municipal home health care sector, which is facing pressure concerning resources because of earlier discharge from hospitals and a growing number of patients receiving medications and treatments at home [7]. Prior studies indicate positive attitudes among decision-makers about eHealth services in municipal home health care, regarding it as an enabler and simplifier [8], which is in line with the political agenda. However, despite its many affordances, many implementations of eHealth services fail [9-11]. This might be because of resistance among health care workers [12], who correlate eHealth with threats to essential care values, fear of lost power, and professional integrity [13]. In a similar manner, eHealth can be experienced by health care professionals as taking focus from patient-centered meetings [14-16] and contributing to an increased amount of documentation and standardization of tasks, with lower staff influence as a result [17]. Thus, attitudes about eHealth seem to differ between policy makers on the one hand and health care professionals on the other hand. Prior studies have shown that the implementation of eHealth services is a complex process that changes health care organizations and the work of health care professionals in both intended and unintended ways [18,19]. To further understand the impact of eHealth and how it influences health care and health care work, extended knowledge about how health care workers experience different health services in different contexts is needed. Most studies regarding eHealth are researched in hospital care, whereas there is an extensive gap in research regarding eHealth services in professional home health care settings, especially services that aim to facilitate and make communication between professionals more effective [20,21]. To gain more knowledge in this field, where the use of eHealth is particularly expanding, this study focused on nurses’ experience of an electronic medication administration record (eMAR) used to communicate and sign delegated nursing tasks in a home health care setting.

### Setting and Technology

In Sweden, a person in need of care at home can apply for assistance. In cases of demand for care such as personal hygiene, food delivery, or purchasing, this is provided by a health care assistant (HCA). On the other hand, medical care is provided by a registered nurse. A customary solution in home health care is for HCAs and nurses to work in different organizations within the municipality. In cases of support for medical care, it is common for nurses to transfer tasks to HCAs through delegation. Delegation is regulated by the National Board of Health and Welfare’s National Guidelines [22]. A frequent intervention in home health care is support regarding drug administration [23], which is also the most delegated task from nurses to HCAs in home health care [24,25]. It has been shown that common deviations regarding drug administration are failure to sign the list after administration, which has traditionally been made manually on paper; incorrect doses; and omission of doses, which can harm patient health [24,26,27]. Studies have shown that the use of an eMAR may increase patient safety and reduce common mistakes [28,29]. Therefore, more than half of the 290 municipalities in Sweden have recently implemented eMARs to improve efficiency and increase patient safety [30]. The eMAR in this study was implemented through the initiative of the local municipal board as part of a municipality-wide eHealth strategy, which is in line with the National Digitalization Strategy in Sweden [31].

The eMAR is used for the documentation of medication administration and other delegated tasks. It contains a worklist generated by the nurse to support HCAs, informing the tasks to be performed for each patient—for example, administer drugs to patient *X* at 8 PM—and the HCA logs the administration of medication manually. The eMAR does not interoperate with the EHR but is a separate system. More particularly, it comprises a web-based administration tool on a computer and a mobile app of the eMAR. Access to the eMAR in the computer is limited to system administrators and authorized personnel (nurses and managers). Through computers, nurses create worklists and handle delegations. The eMAR also provides information and statistics on signed and unperformed interventions at the individual and group levels, information on medication availability in patients’ homes, and information on delegations. When a nurse registers a task via the computer, it is displayed directly in the mobile eMAR app and is visible to the HCA. The eMAR app can be used when nurses perform health- and medical care–related tasks; however, it is mostly used by HCAs when performing delegated tasks, such as drug administration to patients. The HCA creates a signature in the wearable eMAR in connection with the delegated task being performed.

In summary, there is political intention in Sweden and other countries to use and introduce eHealth, and most municipalities are active in this regard. Common eHealth services are eMARs that aim to communicate delegated tasks among professionals. However, more knowledge is needed regarding how the use of eMAR systems is experienced by municipality home health care nurses in their daily work. With nurses being one of the critical users of eMARs, their experiences provide a beneficial understanding of the challenges and profits related to the use of similar eHealth services.

The aim of the article is to shed light on how home care nurses experience eMARs in a Swedish municipality.

## Methods

### Study Design

This paper describes home care nurses’ experiences with an eMAR used in a Swedish home health care setting. A qualitative inductive approach was applied using focus group interviews as the data collection method. The analysis of the interviews was conducted using constructivist grounded theory (GT), according to Charmaz [32,33].

### Setting and Participants

Nurses employed in a municipality in southwest Sweden, where an eMAR has been used since May 2019, were asked to participate in the study. Nurses were responsible for patients in municipal home health care. They were first informed orally about the meaning of the study by their managers and received oral and written information from the study’s authors. Of the 19 day-employed nurses in the municipality, 16 (84%) nurses participated in the study. The authors were informed that the 16% (3/19) of nurses who did not participate in the study were not working when the interviews took place. There was no prior relationship between the participating nurses and the researchers conducting the focus groups.

### Data Collection

Data collection took place from November 2019 to January 2020, using focus group interviews conducted by the first and last author (SK and MNP), 6 and 8 months after the implementation of the digital application. The interviews were accomplished in 4 focus groups, with 5 to 7 nurses in each group, and took place at the participants’ workplace. Approximately 63% (10/16) of the nurses participated in the focus group interviews both 6 and 8 months after the implementation. Furthermore, 13% (2/16) of nurses only participated in the focus groups conducted 6 months after the implementation. In contrast, 25% (4/16) of the participating nurses only participated in the focus group conducted 8 months after the implementation. This arrangement was because of practical reasons, as not all nurses in the municipality were working at the same time. The participants’ average work experience as a nurse in the municipality where the study took place was 11 years, ranging from <1 year to 30 years of employment. Each focus group interview lasted for 60 to 70 minutes and was recorded using a mobile microphone. The discussions in all focus groups began with an initial open interview question. No other interview guides were used. The initial question concerned the participants’ experiences of using the eMAR: *what is your experience working with the digital application?* During the interviews, participants were encouraged to focus on the general topic announced by the initial question. The interviewer asked follow-up questions such as *can you explain* and *in what way* to obtain detailed answers.

### Analysis

Interviews were transcribed and analyzed following constructivist GT, according to Charmaz [32,33]. Charmaz [32] applied a social constructivist approach to GT, which considers reality as diverse and relates to the interviewees’ statements and the researcher’s interpretation of these as one of the many descriptions of reality. Furthermore, a social constructivist approach acknowledges that eHealth services are products made by humans within particular social and historical contexts who bring their understanding and assumptions into practice and, thus, represent and promote social institutions and hierarchies [34-40]. After the first interview, the data were transcribed and read several times by the first and last authors (SK and MNP). Initially, the data were coded line by line, in so-called initial coding, which implies naming each line of written data with a label that categorizes, summarizes, and accounts for each piece of data, emphasizing the actions and processes. The next step in the coding process involved focus coding, which compared the different initial codes, asking which theoretical categories these statements may indicate. Furthermore, the initial codes were assessed using data, and codes with greater analytic power were distinguished into focus codes. This coding process continued during data collection in the following 3 focus group interviews. When all data were collected, focus codes were compared and merged into further abstraction in so-called conceptual categories, covering the core meanings of nurses’ experiences. During the analytical process of the research, analytical ideas that occurred were written in memos, which were informal notes written individually by the researchers. Bearing thoughts in the memos were further developed in the conceptual categories. The coverage of the codes and categories in the data was checked during the analysis, which was then discussed by the authors and experienced GT researchers.

### Ethics

The study was reviewed and approved by the Swedish Ethics Review Authority (Drn 2019-03263). Participants were informed of the voluntary nature of participation and the right to withdraw at any time. The use of the mobile recordings was explained before each interview. Data were handled confidentially.

## Results

### Overview

Nurses’ experiences of eMAR were described in the following two categories: *nurses become monitors* and *slip away from the point of care* with their respective codes. *Monitoring through transparency*, *monitoring shift focus*, and *monitoring changes power relations* were recurring factors in the data related to the first category. Monitoring was possible through the increased transparency provided by the eMAR and the measurands that the nurses also applied, focusing on the quantitative aspects of the delegated nursing tasks rather than the qualitative aspects of the tasks. Furthermore, nurses’ monitoring of the HCA’s work performances through the eMAR affected the power relations between the professions. The second category included the codes *eMAR creates new working tasks* and *eMAR enforces delegation as routine*, as the eMAR entailed an increased amount of administrative work away from the point of care and further strengthened the way of managing work through delegation to HCAs.

### Nurses Become Monitors

#### Overview

The nurses delegated most of the drug administration to HCAs. However, because of legislation, nurses were still responsible for the task being performed correctly. The eMAR allowed nurses to monitor HCAs regarding their performance of the task continuously and was considered by the nurses as giving them insight into the HCAs’ work. Monitoring was enabled through the enhanced transparency afforded by the eMAR service. However, it also appeared that the monitoring prompted nurses to focus on the quantifiable parts of the working tasks as they applied the eMAR’s specific focus on time, the performer, and its measurable variables about the delivered care. In this way, the nurses experienced monitoring as causing a shift in focus from other aspects of the task. In addition, the monitoring affected the relationships with HCA, where the nurses’ superior position was considered to be strengthened because of the monitoring possibilities.

#### Monitoring Through Transparency

The eMAR provided transparency for nurses through real-time information about the HCA’s performance regarding delegated tasks. In this way, nurses were able to control and keep HCAs under surveillance. When the eMAR was introduced, nurses realized that delegated tasks were not always performed correctly. The nurses estimated that the number of missed tasks was much greater than before digitalization, as they had rarely noticed omissions before. Transparency meant that the nurses could act on the information given by the eMAR immediately, something they regarded as positive, as they felt responsible for the delegated tasks being performed correctly. The increased information provided by the eMAR was considered positive in terms of patient safety and quality of care, as the nurses now had the possibility to acknowledge mistakes and work on improvements:

We see everything that happens. Now we notice how much is not going right, we did not do that before.

Before eMARs, signing lists were usually collected for inspection by the nurse every 1 or 2 months. With eMAR, nurses experienced reduced paper administration as they did not need to check and change documentation at the patients’ homes. This work could now be conducted from the office. The ability to discover deviations and update information at a distance from the office was regarded as positive.

#### Monitoring Shifts Focus

The scope of the use of the eMAR varied among nurses. It replaced the paper-signing lists; thus, all nurses were obliged to use eMAR to document and control the delegated tasks. However, some inscribed functions (built in by designers) were only used by a small number of nurses; for example, the ability to compare the number of alarms within and among groups of HCAs. Furthermore, nurses assessed information in diverse ways. Some estimated the documented information in eMAR as neutral facts, whereas others perceived it as containing a correct but reduced part of reality, which had to be complemented with other sources of information. Sometimes, the HCA performed the medication administration correctly but failed to document it properly.

The eMAR provided information about the time at which the drug administration had been performed and by whom. However, qualitative aspects of the performance, such as whether the patient was given the correct information, critical estimation about the plausibility of the prescription, patient involvement, the quality of the performance, or other aspects of nursing work, were not included in the information provided. However, most nurses used this reduced quantified information as a basis for assessing how well the HCAs performed their work. This resulted in quantification of HCAs’ work performances, as the nurses applied the apps’ specific focus on time and documentation:

And you can get a report for each specific HCA as well as what percentage they are on, so you can see if it is one that has hundred percent or if someone has only signed 80 percent in time, then maybe you should have a conversation with that person.

The inscribed functions and design of the eMAR also influenced the focus of the users. Nurses experienced that HCAs used the information in the eMAR to a greater extent than the information that was still provided on paper. For example, when administering drugs, one must check the medication, identity of the patient, written medication list, and information in the eMAR. However, the nurses experienced that HCAs did not always check the medication list on paper and relied only on the information in the eMAR. The nurses thought that this was because the information was stored in separate places, and the technology was accessible and more attractive than paper to use. According to the nurses, HCAs’ prioritization of performing the tasks included in the eMAR sometimes led to a decreased focus on other important areas of their work, which were not included in the eMAR. As the following quote illustrates, the focus shifted from the patients to the eMAR and documentation itself:

When I worked one weekend, I remember it very clearly, they were so stressed. There was a member of staff there and she was sweating, she just focused on the app “There mustn’t be any delays, mustn’t be any delays.” And I was just: “But you will not be hanged because you have given a drug five minutes late to a patient.” But she was really worried about that she would get rid of her delegation to give drugs. It was the only thing she focused on, to give drugs within the time-frame in the application, and the risk is that she missed something else, like how the patient in front of her was doing.

Correct documentation could also give nurses a false picture of everything working well in care situations. Only when measurable tasks within the eMAR were omitted were the nurses informed. In the case of other misconducts, eMAR did not provide any information.

#### Monitoring Changes Power Relations

The eMAR offered an opportunity for nurses to control the HCAs’ work performances. Monitoring affected their relationship with HCAs, changing the power relations between the professions. HCAs could not know when or if they were being checked. Nurses felt that the monitoring possibilities sometimes created stress, fear, and frustration among HCAs, and they described increased power in relation to HCAs. The eMAR alerted the nurse with visual alarms when a task was not performed correctly, which led to a focus on negative feedback from the HCAs. Monitoring was perceived as a new and complex situation for nurses:

It is a shift of power in that we can get such control over what they do. And they know about it. In that way, it is a power factor. That’s exactly what it is, you go in and say “Now I have seen this mistake, so now we withdraw your delegation...so they are a bit stressed sometimes.”

Although the eMAR was implemented as an initiative from the local municipal board, the nurses felt that they were held accountable for the implementation by the HCAs, who were frustrated with the nurses’ monitoring and focus on documentation:

It is negative stress. Sometimes it feels like they think we have invented this. “How silly, we do the best we can, and we don’t have time and we are so stressed,” and so on. Then we have to promote this eMAR and explain that it is a quality system that we use according to our guidelines. Sometimes I feel that we have to take the shit for this in some way.

### Slip Away From the Point of Care

#### Overview

In addition to the monitoring illustrated in the category above, the nurses also experienced that their professional role was transitioned, as the eMAR imposed them to perform new administrative working tasks and was used and designed to facilitate delegation as a modus operandi in the home health care organization. This further positioned nurses to administer and control nursing tasks rather than perform the tasks themselves.

#### eMAR Creates New Working Tasks

The implementation of the eMAR brought new assignments and areas of responsibility for the nurses, such as investigating deviations to a much larger extent than before, having personal follow-up meetings with HCAs, and providing technical support to colleges. Although they were grateful for the reduced paper administration because of digitalization, they also experienced an increased amount of these other administrative tasks, which they regarded as time consuming and not a part of what they considered their actual responsibilities as nurses. For example, the eMAR often alerted when a task had not been signed at all, and to investigate such a situation, the nurse had to find out who was responsible for performing the task among the HCAs. They needed to ask the HCA unit manager for information regarding work schedules, which often required emailing and calling to get hold of information. Notifications from the eMAR demanded immediate action from the nurses not only s they were responsible for the delegated tasks but also to keep the mobile screen for HCAs clean; otherwise, it would have been impossible for them to see other relevant information behind the notifications. The need for prompt action on eMAR alarms resulted in workflow disruptions for nurses. They felt that they were spending considerable time in investigations and personal follow-up conversations with HCAs and assessed this type of work as being outside their nursing profession and actual responsibilities:

And at the same time, you have all your own work that you have to keep up with and you sit there in conversations with managers and healthcare assistants, yes, it is not entirely simple. It can be quite frustrating when you have the whole diary full, and you have three booked meetings where you must discuss this and try to be flexible and still clear about the rules. 

The nurses were not involved in decisions regarding the implementation of the eMAR and had no established formal ways of providing feedback to the eMAR developers. Smaller updates and new functions came without prior notice, and as the delegated tasks were considered to be the nurse’s area of responsibility within the organization, HCAs turned to them with questions about technology and its use:

They come and ask me “How do I do this in the application, how does the application work?”...Yes, you feel that you are not only a nurse but also have become some kind of technical support or something. For my part, it’s all right, but it’s not really part of my job as a nurse (laughs).

Nurses expressed that they had the skills and abilities necessary to use digital health technology and services. They had experience working with eHealth services as EHRs, digital booking systems for medical products, mobile telephones, and email services were already a part of their daily work. The fact that not all systems were integrated was something they perceived as time consuming because of different log-ins and the occasional need for double documentation. The nurses expressed ambivalence about the functions of the various systems but accepted the organization’s existing digital systems, although they did not always facilitate their work:

You just have to teach yourself and become friends with the systems...I don’t know, it is difficult to dislike something that we must use. You just have to learn.

#### eMAR Enforces Delegation as Routine

The nurses’ experiences indicated that the delegation of drug administration was routine within the organization. There were not enough nurses employed to administer drugs to the patients themselves without delegating the task to HCAs. Nurses said that this situation also existed before implementation. However, eMAR was designed in accordance with this organization of work and, in this way, strengthened delegation as a way of working. The nurses felt a certain pressure to delegate the administration of drugs to HCAs:

Quite simply, we have to deliver a delegation to someone we have barely seen or know who they are or what they are capable of.

The nurses had formal responsibility for medical tasks even if they were delegated, and they tried to find ways of influencing the HCAs’ ability to fulfill these tasks correctly. This was realized by providing suggestions about the changed working routines in the organization. However, the nurses experienced a lack of mandate to influence organizational issues, and their attempts to organize the HCAs’ work were not encouraged within the organization:

It’s not so simple. If I say: “The two who have the phones with the application must be responsible for the drug administration,” then I am perceived as controlling how they should do their work, and then it does not fall on fertile ground, and I get back from the management “That it is not my job to tell how they should do it.” But the reason I do it is that no one else does it either. I have a responsibility to ensure that the drugs are given when they are supposed to be given, and if no one else takes responsibility for personnel management, you must go in and give a suggestion.

Owing to a perceived lack of mandate to influence identified structural causes of deviations regarding the delegated tasks, such as understaffing, lack of medication competence among HCAs, lack of internet connection at some places in the municipality, provision of portable devices that enable mobile connection for the eMAR, or issues regarding work organization, the nurses focused on individual causes of deviation, which was supported by the organization’s guidelines. The individual focus on personal follow-ups with HCAs was experienced as frustrating as nurses sometimes felt that the discussions made no difference.

## Discussion

### The eMAR Has Transformative Effects

eHealth has many affordances, and it is important to acknowledge its many possibilities. The expectations for what eHealth can offer are often high, which is indicated in the very adoption of the concept, as defined and extensively cited by Eysenbach [1] as follows: not just a technical development but also an attitude that the use improves health care locally and worldwide. This enthusiastic state of mind is also visible in the Swedish Governmental Vision of eHealth 2025 [31], which aims for the country to be the best worldwide in using the opportunities offered by digitalization. However, it is not surprising that digitalization in health care is proceeding at a rapid pace. However, this study shows that the use of the eMAR was experienced as transforming some aspects of the nurse’s profession toward a function of more control through monitoring and administration. This was not a pronounced goal for the use of eMAR but instead, an unintended consequence that might add to the understanding of the different views of eHealth between policy makers and health care professionals that now exist. The findings in this study are in line with results from previous research, stating that nurses considered documentation and navigation in the eMAR system as time consuming and took time away from their patient near work [16]. However, previous research has also stated that using an eMAR does not result in less direct patient care [41]. Consequently, this study shows that a broad perspective should be considered in eHealth interventions, embracing both the intended and unintended aspects of their meaning and impact on social relations, professionals, and organizations. Therefore, these impacts should be taken into consideration when planning for and using this type of eHealth service.

As we have shown, the eMAR enabled nurses to monitor HCAs in real time regarding delegated tasks. The monitoring resulted in increased insight and clarity for the nurses, which was considered positive concerning patient safety, as they experienced more control over their area of responsibility. However, the information provided by the eMAR was mediated through its measurands, which focused on time and the performer, only making specific parts of reality visible. In this way, the use of the eMAR prompted nurses to assess tasks through numbers and measure the HCA’s work performance in percentages, which they regarded as a focus shift from more qualitative aspects of nursing. This process can be understood using literature on transparency related to visualizing technologies. Flyverbom [42] uses the term *prism* as a metaphor for transparency to illustrate how visualizing technologies provide a mediated representation of reality. In this way, transparency not only makes certain things visible but also produces differences [37]. Orlikowski and Scott [43] made a similar reasoning, stating that materializations of discourse have performative consequences that affect practice. In our case, before the use of the eMAR, signing lists for documentation of medication administration were materialized in paper sheets in the patient’s home. In contrast, the eMAR provided nurses with information about the person who performed the delegated task and the time when it was performed. Using the Orlikowski and Scott [43] perspective, this is most likely a material-discursive shift from retrospective transparency to real-time transparency, which resulted in work disruption and new working tasks for the nurses who had to act on the information by investigating deviations and holding follow-up meetings with HCAs. Real-time monitoring also affected the power relations between the 2 professions, strengthening the HCAs’ subordinate positions.

Furthermore, the transition of the nursing profession toward a function of controller, as well as an increased focus on administration and measurands, is in line with the New Public Management model, introduced in the Swedish health care sector in the 1980s [44], where the pursuit of control, efficiency, productivity, and transparency is fundamental. The eMAR might further strengthen this development. However, depending on its design, an eHealth service may rely on, develop competence and judgment, or foster quality improvement through control, review, and governance*.* It is not implausible to consider that using an eMAR, where the measurable is prioritized, may lead to a focus on measurable parts of health care tasks, in favor of more immeasurable tasks, for example, to stop talking to patients while a task is performed and relieve anxiety by listening or the use of personal judgment.

### eMAR Enforces Delegation as Routine

Our findings demonstrate that nurses experienced delegation as praxis within the organization and that the eMAR supported this way of working. The nurses felt that they were expected to delegate tasks such as drug administration to HCAs. The Swedish National Board of Health and Welfare’s regulations and general advice on the delegation of tasks in health care [22] describe that delegation can be used in exceptional cases and must not be applied to solve staff shortages or for economic reasons. Nevertheless, former studies have shown that HCAs perform most tasks requiring nurses, and instead of being a complementary solution, where medical tasks such as drug administration are undertaken by nurses, delegation has become routine in Swedish home health care settings [24,45]. This was confirmed by this study’s results.

The study findings revealed that the eMAR alerted nurses that existing structures regarding delegation within the organization did not always function properly. Nurses experienced an increased omission of doses and late administration of drugs after the implementation of the eMAR. Medication administration is a complex task requiring counting, mixing, calculating, and controlling that the administration be performed at the right time for the right person in a correct dose and for accurate purposes [46]. The eMAR only provided information on parts of the task, leaving out other aspects of the administration. Even if the nurses experienced that their increased control of medical administration through the eMAR had positive effects on patient safety, they also acknowledged that other aspects that were not visualized in the eMAR had lower quality. The nurses’ experiences of omissions regarding drug administration and other commonly delegated tasks should be further investigated to gain more knowledge about how this form of extensive delegation of medical tasks to HCAs affects patient security and quality of care.

To improve the situation regarding omissions, the nurses proposed structural changes in how HCAs worked. However, as they felt a lack of mandate to influence organizational issues, they focused on having follow-up conversations with individual HCAs. This was regarded as a time consuming and complex task to accomplish, partly because of the negative character of their feedback to the HCAs. This complexity regarding reporting and communicating adverse events in home health care has been highlighted in a prior study demonstrating that reporting of adverse events that involve colleagues is considered distressing by nurses [47]. The eMAR used in this study allowed nurses to observe inaccuracies in delegated tasks. To use this information constructively so that future mistakes can be prevented and a culture of safety may be fostered in the health care organization, structures for processing this increased amount of data are needed. Focusing on individual feedback rather than structural ones might hinder organizational changes that can contribute to delegated tasks being performed correctly. Therefore, we suggest that the information regarding mistakes and deviations displayed in the eMAR should be analyzed and managed with a broader focus than on individuals and at a higher level within the organization so that preventive work and a structural take on deviations can be conducted.

eHealth services were not new to the participants in this study. Several eHealth systems were already in use when the eMAR was implemented. Experiences from prior eHealth services, as well as eHealth services in use, might have influenced the nurses’ experience of the eMAR. For example, previous research highlights the connection between the general use of digital technologies in daily life and the perception of digital technologies at work, stating that people who appreciate the advantages offered in a digitized society also recognize and expect profits provided by digital technologies at their job [48]. However, these aspects were not investigated further in this study.

### Co-design as a Possibility

The findings in this study also indicate that the eMAR was implemented from the top of the organization and that the principles of co-design were not applied. Nurses had many ideas about the desirable features and design of the eMAR but lacked formalized paths to communicate with eMAR developers. The sender was regarded as unclear, and smaller updates were unannounced for reasons that the nurses were not informed of. The impression that decisions about eHealth services are made by unidentified persons at a higher level in the organizational hierarchy indicates a lack of employee involvement and has been previously acknowledged in research concerning eHealth in health care organizations [49]. As the eMAR was dependent on internet access and did not work in all geographical locations in the municipality, security was not perceived by the nurses to meet the safety requirements of health care. Therefore, analog paper copies and backup plans were always available. This suggests that the eMAR was not fully adapted to a specific context. Acknowledged factors for successful implementation of eHealth services are user involvement before implementation, sufficient time for users to learn the system, formalized feedback sessions where users can discuss experiences and specific issues, and available support from super users of the implemented system within the workplace [8,50]. Furthermore, the interaction between users and developers should continue, as problems and challenges in the system are addressed once the eHealth service is in use [36,51]. Therefore, we suggest that future implementations and uses of eHealth services involve end users to ensure alignment with the ongoing processes in the health care organization, as well as to incorporate professional values and understandings. Through collaboration, nurses and other health care professionals might have a greater influence on which areas of their work can benefit from eHealth and in what direction their professions will develop.

### Methodological Discussion

Using GT analysis, according to Charmaz [32,33], proved to be useful in exploring how home care nurses in a Swedish municipality experienced eMARs. This method was considered helpful in achieving the objectives of the study. Furthermore, using interviews for data collection made it possible to see diversity in reality; however, there were some aspects that must be emphasized. First, the number of participants in the focus groups is recommended to be small [52], which was the case in this study, so that all the participants are able to speak. The composition of the group, in accordance with Krueger and Casey [52], was homogeneous, as all participants were nurses working in the same organization. Familiarity with technology is related to improved acceptance, and some challenges might only be visible during the first months immediately following implementation [53]. The dates of the focus groups were set so that participants would have some experience with the eMAR and, at times, intended to make participation possible for most of the nurses employed in the municipality. For practical reasons, some nurses participating for the second time, 8 months after the implementation, were placed in the same group as nurses who participated for the first time. Whether the composition of the focus group interviews influenced the discussions at the time of the interview and, thus, the results is difficult to say. On the other hand, the number of nurses in the municipality where the study was conducted was small, and almost everyone (16/19, 84% nurses) participated. Therefore, there is reason to believe that nurses discussed the issue on occasions other than during the interviews. From this point of view, the result is not believed to have been affected by the fact that some nurses participated for the second time, together with those who were interviewed for the first time. Second, to achieve credibility [54], the analysis was conducted jointly by the first and last authors (SK and MNP). The findings have also been discussed in 2 different seminars with other researchers affiliated with nursing and sociology. Finally, the process in this study is described in such a way that it can be followed and repeated by others. However, the findings in future studies may differ as the use and development of technology is rapidly advancing, and the user adapts to prevailing conditions. The nurses participating in the study were offered to take part in the interview transcripts but did not ask for it.

The social constructivist perspective applied in the study distinguishes itself from the assumption about objective reality and, instead, adopts the assumption that reality is multiple, processual, and constructed [32,33]. In this manner, the study did not aim to evaluate the efficacy of the eMAR; rather, the experiences of its users and the human and technology interactions were explored. When adopting a social constructivist perspective that recognizes eHealth services as social artifacts, it is possible to highlight the intended and unintended consequences and implications on existing hierarchies and social life. The constructivist approach has been useful in acknowledging this broader experience of eHealth services and recognizing larger structures embedded in participants’ experiences.

Future studies should focus on the attitudes and experiences of end users of eHealth services, as well as developing theories and methods for investigating the effects and processes when technological and human agency come together in health care.

### Conclusions

Previous analyses of eHealth services in health care have shown that implementation is a complex process that changes health care organizations and the work of health care professionals in both intended and unintended ways. This study adds to the literature by examining how users of a specific eHealth service—an eMAR used to manage the delegation of medical administration—experienced impacts on their daily work. The results showed that the nurses experienced that eMAR gave them more control and knowledge about how delegated tasks were performed. However, the eMAR also brought more complex and unexpected changes to their work situation, such as more administration, a focus shift toward quantifiable aspects of work, and changed power relations between the professions. As the nurses applied the eMARs’ specific focus on time and documentation and its broader focus on delegation as a way of working, the results also indicate that inscribed functions in an eHealth service may affect values and priorities where the service is in use. This presents an opportunity for future research to assess the impact of specific eHealth services on health care professionals’ work and further examine the effects of inscribed functions in relation to how they may affect actions and priorities at the individual and organizational levels.

## Abbreviations

EHR: electronic health record
eMAR: electronic medication administration record
GT: grounded theory
HCA: health care assistant
